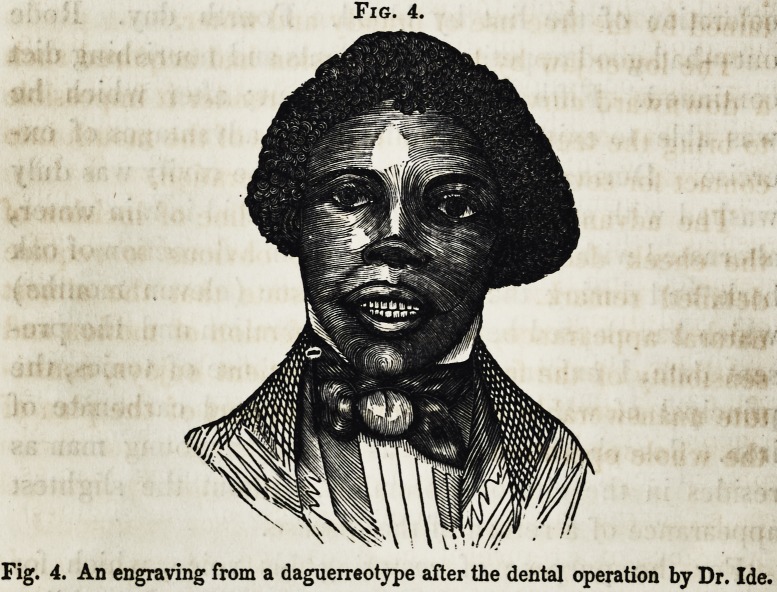# Resection of the Left Superior Maxillary Bone

**Published:** 1850-04

**Authors:** R. Thompson


### ARTICLE V.

*Resection of the Left Superior Maxillary Bone.* By R. THOMPSON, M. D.

FIG. 1.

Fig. 1. An engraving from a daguerreotype before the operation.

The subject of this operation, Joseph Day, a young man, aged twenty years, about five years ago had an attack of pain in the second molar tooth of the left superior maxillary bone, which he believed to be nothing more than ordinary tooth-ache. The tooth, soon becoming loose, was removed; after which, within a very few days, he discovered, protruding from the place previously occupied by the tooth, a small projection or tumor, which, though it grew but slowly, was deemed of sufficient importance to demand attention. His parents accordingly consulted, from time to time during the progress of the case, several physicians, without obtaining any important relief, or definite opinion as to its nature. During the first three years of its history, the growth of the tumor was gradual—its direction of development being forward and lateral, occasioning the loosening and removal in succession of all the teeth in the line of its progress, and extending itself to the right so as to occupy a considerable portion of the cavity of the mouth, while its outer enlargement somewhat distended the left cheek.

Such, so far as I have been able to ascertain, was the state of the case when the young man visited Columbus for surgical aid, in the summer of 1847. Not having received much encouragement as to the object of his visit, he entered a boarding-house as a servant, in which capacity he continued until the meeting of the Med. Con., May, 1848, when he presented himself before said body, with a hope that he might yet find relief.

The tumor having grown very rapidly during the preceding three months, it then (when I first saw the patient,) filled to its entire capacity the cavity of the mouth, separating the jaws to the distance of an inch, protruding between the teeth, greatly distending the left cheek, involving in its substance the greater part of the left maxillary malar and palatine bones, filling and greatly distending the maxillary sinus, and displacing to the right the nasal septum, and presenting in its general aspect a case so formidable as to assure every intelligent observer that death would soon close his career, unless deprived of his victim by surgery.

To the eye, that portion projecting between the lips presented the appearance of fungus hæmatodes, while the touch discovered in it a greater degree of resisting solidity than is ordinarily found in developments occasioned by that terrible disease. Nor did the tumor manifest the disposition to hemorrhage so common in malignant fungus—having never proved troublesome or dangerous in this regard with the exception of a profuse bleeding which resulted from an exploring puncture made by a lancet several months before I saw him.

His bodily health, which till within a few months had been tolerably good, was now failing, partly from the irritation of the disease, but mainly from the want of sufficient nourishment, as he was deprived of the power to masticate a morsel of solid food, and even of the ordinary enjoyment of the liquid aliment, upon which he was obliged to subsist.

From the foregoing considerations, together with the fact that during its progress the patient had experienced but little pain, I came to the conclusion that the disease presented more of the characteristics of osteo sarcoma than of any other morbid growth which had ever fallen under my observation.

Under such circumstances, though sustained in opinion as to the result of the operation by but few, I determined to afford him the only chance now remaining for his life. The patient was anxious to live, and willing to hazard an operation, though the chances of ultimate recovery, as he was informed, stood in fearful odds against him. I agreed to operate, and with the result, as here presented, I have reason to be satisfied—a living, healthy, and but slightly marred young man.

OPERATION.—On the first day of July, 1848, all necessary preparations having been made, I proceeded to the operation in the presence of several professional gentlemen and other citizens.

The patient was placed upon a barber's chair, with his head thrown obliquely back upon his right shoulder, and supported by an assistant. With the scalpel in the left hand, (standing behind the patient,) I made a straight, deep incision, from the angle of the mouth to a point, midway between the external angle of the eye and the ear.

Two arteries, (a branch of the internal maxillary and the facial being divided,) were secured by ligature, after which the anterior flap was hastily dissected up to the middle of the nose, and cutting up the cartilages of the left wing of the nose, after which the posterior soft parts external to the tumor were dissected backwards and around the maxillary bone to its pterygoidal attachment to the sphenoidal bone.

The tumor externally being perfectly exposed, it was deemed proper to make the superior section of the bone in a transverse line below the infra orbitar foramen, by which the most prominent points of the os malla, with its zigomatic and internal processes, the floor of the orbit, and the greater part of the nasal process of the maxillary bone would be preserved—parts which more than any other make up the outline, and give definite configuration to the face. The division as thus indicated, (and represented in fig. 3,) was effected by means of saws.

The next step in the process was the section of the bones in the perpendicular line of the face, which was effected by introducing a strong, narrow-bladed saw into the nasal termination of the transverse section, with its points directed towards the throat, by which I was enabled at once to divide the maxillary and palatine bones with the soft parts of the tumor which lay within the range of the instrument. Next in order I separated the maxillary bone from its pterygoidal attachment to the os sphenoides. This was readily effected by the introduction of a narrow, strong, curved spatula between the bony processes, by means of which a wrenching movement was made with the right hand, while with the left the mass was depressed and effectually detached from its long connection. I then divided with a curved knife all visible attaching soft parts, that I might be enabled to bring into view and separate the vellum palati, which was readily done by exerting a forward and downward gentle traction upon the tumor with the left hand, while with the knife in the right, this part of the operation was completed.

That part of the tumor which occupied the right side of the mouth, was attached to its roof by fibrous union, and was readily detached by means of a flat hook, which was introduced behind the remaining mass.

Fig. 2.

Fig. 3.

Fig. 2. An engraving from a daguerreotype after the operation.
Fig. 3. Shows the sections of bone.

The operation being thus far completed, the wound was examined, and to certain points of questionable appearance actual cautery was freely applied—the cavity inlaid with slips of patent lint, wetted in a decoction of white oak bark saturated with alum—the incision closed by the twisted suture and adhesive strips, made by the solution of gutta percha in chloroform, and the patient laid in bed, having been upon the operating chair twenty-eight minutes. Took nourishment and stimulants during the day—slept well during the night. Second day. The wound in the cheek having united by the first intention, I removed the needles twenty-five hours after the operation. Patient took food and stimulants—slept well during the night. Third day. Removed the lint from the cavity. Not the slightest discoloration of the lint by blood. Fourth day. Rode out—had good appetite—stimulants and nourishing diet continued. Fifth day. Walked out; after which he was able to enjoy the comforts and advantages of exercise. During the healing process, the cavity was duly washed with a weak solution of nitric acid in rain water, alternated with the solution of alum in a decoction of oak bark, and during the interval of time (eleven months) which has elapsed between the operation and the present date, I enjoined upon him the use of tonics, the principal of which was the precipitated carbonate of iron. Joseph Day is now as healthy a young man as resides in the city of Columbus, without the slightest appearance of a return of the disease.

For the purpose of restoring his voice, which for several months had been very imperfect, and enabling the patient to take food and drinks with a greater degree of comfort, I, on the tenth day after the operation, fashioned of gutta percha, and introduced into the mouth, a temporary jaw and roof, which answered every anticipated end, until the parts were deemed sufficiently contracted and firm to admit of the adaptation to the parts of a metallic palate and porcelain teeth, which were devised and constructed in a most masterly manner by one of our excellent dentists, Wm. E. Ide, M. D., of this city, whose skill and execution have restored to the individual the power of mastication on the left side of his mouth, and improved his voice so as to leave a stranger unadvised of his previous misfortune, while it assures us of the importance, in view of both the utility and beauty, of the highly improved profession of Dental Surgery.

Fig. 4.

Fig. 4. An engraving from a daguerreotype after the dental operation by Dr. Ide.

Remarks.—Before entering upon the operation, I informed my patient of my intention as to the use of chloroform—stated that my purpose was *not* to induce a perfect suspension of consciousness, but to have him in such a state as would enable him to free his throat from the blood which would unavoidably flow into it; while his sensibility would be so suspended as to enable him to endure the operation with comparatively little suffering; and so attentive was he to the suggestion, that he did frequently spirt the blood violently from his mouth, and occasionally as his sensibility returned, called for "more chloroform," so sensible was he of its anæthetic efficacy.

Two arteries only required the ligature, as before stated; yet capillary hemorrhage was so profuse as to somewhat embarrass the operation, and considerably prostrate the patient, whose strength was greatly sustained by the free use of brandy and water.

The lower jaw by long depression had acquired such a downward curve and twist as to render it impossible to bring the teeth upon the right side of the mouth into contact for several weeks after the operation.

The advantages derived from the line of incision of the cheek decided upon are too obvious to require detailed remark. Suffice it to state that the almost natural appearance, with the perfection of motion and sensibility of the face which the patient enjoys, constitute unanswerable arguments in support of the plan of the whole operation.

## Figures and Tables

**Fig. 1. f1:**
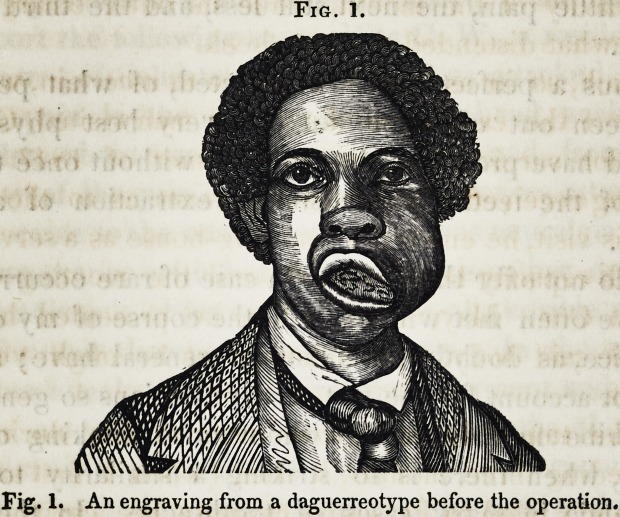


**Figure f2:**
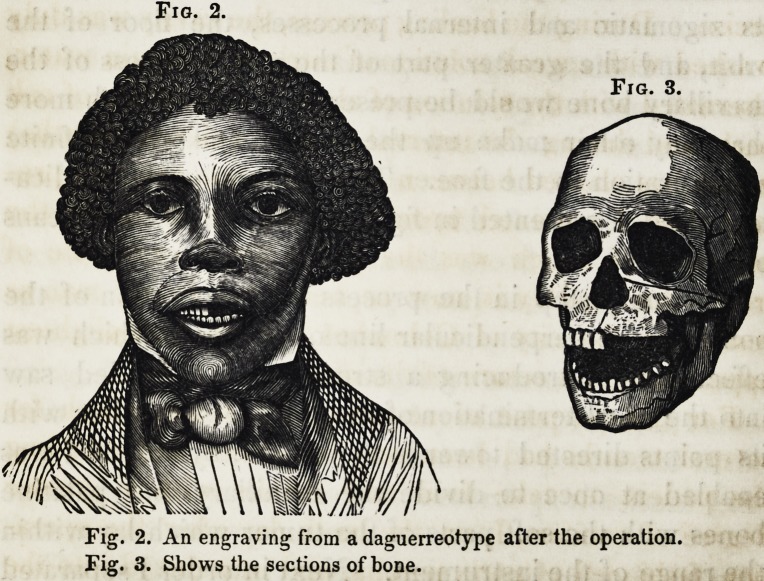


**Fig. 4. f3:**